# The Relationship between Diabetes-Related Complications and Obstructive Sleep Apnea in Type 2 Diabetes

**DOI:** 10.1155/2018/9269170

**Published:** 2018-03-07

**Authors:** Nantaporn Siwasaranond, Hataikarn Nimitphong, Areesa Manodpitipong, Sunee Saetung, Naricha Chirakalwasan, Ammarin Thakkinstian, Sirimon Reutrakul

**Affiliations:** ^1^Division of Endocrinology and Metabolism, Department of Medicine, Faculty of Medicine, Ramathibodi Hospital, Bangkok, Thailand; ^2^Division of Pulmonary and Critical Care Medicine, Department of Medicine, Faculty of Medicine, Chulalongkorn University, Bangkok, Thailand; ^3^Excellence Center for Sleep Disorders, King Chulalongkorn Memorial Hospital, Thai Red Cross Society, Bangkok, Thailand; ^4^Section for Clinical Epidemiology and Biostatistics, Faculty of Medicine, Ramathibodi Hospital, Bangkok, Thailand; ^5^Division of Endocrinology, Diabetes and Metabolism, Department of Medicine, University of Illinois at Chicago, Chicago, IL, USA

## Abstract

This study explored the relationship between obstructive sleep apnea (OSA) and the presence of any diabetes-related complications in type 2 diabetes and whether this was mediated by hypertension. Secondly, the relationship between OSA severity and estimated glomerular filtration rate (eGFR) was investigated. A total of 131 patients participated. OSA was diagnosed using a home monitor, and severity was measured by apnea-hypopnea index (AHI) and oxygen desaturation index (ODI). OSA was found in 75.6% of the participants, 40.5% with moderate-to-severe degree. Any diabetes-related complications (retinopathy, neuropathy, nephropathy, or coronary artery disease) were present in 55.5%, and 70.2% of the participants had hypertension. Mediation analysis indicated that, compared to those with mild or no OSA, those with moderate-to-severe OSA were 3.05 times more likely to have any diabetes-related complications and that this relationship was mediated by the presence of hypertension. After adjusting for confounders, ODI (*B* = −0.036, *p* = 0.041), but not AHI, was significantly associated with lower eGFR. In conclusion, moderate-to-severe OSA was related to the presence of any diabetes-related complications in type 2 diabetes, and the relationship was mediated by hypertension. The severity of intermittent hypoxia was associated with lower eGFR. Whether OSA treatment will delay or reduce diabetes-related complications should be investigated.

## 1. Introduction

Obstructive sleep apnea (OSA) is a common sleep disorder, characterized by repetitive episodes of upper airway closure or partial collapse during sleep, resulting in intermittent hypoxia and fragmented sleep. OSA is a recognized risk factor for insulin resistance and type 2 diabetes (T2DM), independently of body mass index (BMI) [1]. In well-designed laboratory experiments, intermittent hypoxia and fragmented sleep resulted in increased insulin resistance without an adequate compensatory insulin response, leading to glucose intolerance [2, 3]. Furthermore, a recent meta-analysis of over 60,000 participants from nine prospective cohort studies revealed that OSA was associated with a 35% increase in the risk of developing T2DM [1]. While obesity is a well-known risk factor for OSA, there are additional risk factors such as abnormalities in craniofacial and upper airway structure, particularly in Asian populations [4]. Furthermore, muscular dystrophy or other neuromuscular diseases can contribute to obstructive events when presenting as pharyngeal weakness or bulbar involvement [5]. Untreated OSA may impair daytime function and is associated with an increased risk of psychiatric symptoms such as mood or anxiety disorders [6]. The estimated overall prevalence of OSA has substantially increased, and it is highly prevalent in patients with T2DM, between 58–86%, depending on the study population [7, 8], particularly in very obese patients with T2DM [9]. In patients with T2DM, OSA severity has been shown to be associated with poorer glycemic control [10, 11]. Despite the strong association between OSA and glucose metabolism, the effects of continuous positive airway pressure (CPAP) treatment on glycemic control in those with T2DM have been mixed, with some showing favorable effects while others did not [12, 13]. Baseline glycemic status, study design, CPAP compliance, and duration of use may all play a role in these differing results [1].

Poor glycemic control in the long term contributes to diabetic micro- and macrovascular complications [14, 15], resulting in increased morbidity and mortality. Recent data have suggested that the presence and severity of OSA may pose an additional risk factor for these complications [16–19]. Vasoconstriction and increased oxidative stress as a result of intermittent hypoxia can lead to endothelial dysfunction and microvascular impairment [20]. Additionally, activation of the renin-angiotensin-aldosterone system (RAAS) in OSA patients could facilitate the development and progression of diabetic kidney disease (DKD) [21]. In a meta-analysis of 7 studies, OSA was associated with a 59% higher risk of DKD [16]. Moreover, a recent longitudinal cohort study of patients with T2DM, followed for a median of 43 months, found an independent association between OSA and the risk of progression to advanced diabetic retinopathy [18]. Thus, these early data highlight the role of OSA in diabetes-related complications.

It is well known that OSA, especially moderate-to-severe degree, is strongly associated with hypertension [22], and hypertensive patients with OSA are at a higher risk for adverse cardiovascular events [23]. Hypertension also plays a crucial role in diabetes-related complications; systolic blood pressure has been shown to independently and additively exert effects on micro- and macrovascular complications in T2DM patients, in addition to glycemic control [24]. It is likely that hypertension at least partly mediates the relationship between OSA and diabetes complications.

Therefore, the purpose of this study was to investigate the relationship between OSA and diabetes-related complications in T2DM patients and explore whether this relationship was mediated by hypertensive state. Mediation analysis was employed to assess the direct and indirect effect (through hypertension) of OSA on these complications. Secondly, it was explored whether OSA severity was an independent predictor of kidney function, as measured by estimated glomerular filtration rate (eGFR), in T2DM.

## 2. Methods

Nonpregnant adults with T2DM without previously diagnosed OSA being followed at three hospitals in Thailand were invited to participate. The participants were a part of previously described cohorts [25, 26]. Exclusion criteria included those with a history of congestive heart failure or low ejection fraction, chronic obstructive pulmonary disease, end-stage renal disease (ESRD), severe chronic liver disease (such as cirrhosis), stroke, permanent pacemaker placement, or use of certain medications (opioids/narcotics, alpha-adrenergic blockers, clonidine, methyldopa, and nitroglycerin), in order to obtain valid results from the OSA diagnostic method utilized (see below). All participants gave written informed consent. The protocol was approved by the Ethical Clearance Committee, Faculty of Medicine Ramathibodi Hospital, Bangkok (ID 11-60-95).

### 2.1. Assessment of Baseline Characteristics, Glycemic Control, and Diabetes Complications

Weight and neck circumference (cm) were measured. Height, age, current medications (i.e., antidiabetic medications, statins, angiotensin-converting enzyme inhibitor (ACEI), or angiotensin receptor blocker (ARB)), and most recent hemoglobin A1c (HbA1c) values (within three months) were extracted from patient medical records. HbA1c is an index of glycemic control over the preceding 90 days. Body mass index (BMI) was calculated as weight (kg)/height (m^2^). Hypertension was defined as having a clinical diagnosis of hypertension or receiving antihypertensive medication.

Diabetic complications were assessed by interviews and medical record reviews. A history of diabetic retinopathy was obtained from ophthalmologic examinations (within one year) and categorized into those with retinopathy versus no retinopathy. Diabetic kidney disease (DKD) was defined as an estimated glomerular filtration rate (eGFR) < 60 mL/min/1.73 m^2^ (calculated using the Chronic Kidney Disease Epidemiology Collaboration equation) and/or presence of albuminuria ≥ 30 mg/g of creatinine or overt proteinuria, except in 3 patients for whom albuminuria was defined as the presence of albuminuria ≥ 30 mg. Peripheral neuropathy was defined as the presence of symptoms of peripheral neuropathy such as pain or burning sensation. Presence of coronary artery disease was obtained from an interview and confirmed by a medical record review. Presence of any diabetes-related complications was defined as having one or more complications.

### 2.2. Assessment of OSA

OSA was diagnosed utilizing an in-home overnight monitoring device, WatchPAT 200 (Itamar Medical, Caesarea, Israel). This is a noninvasive home sleep test device for detecting peripheral arterial tone (PAT) by a signal that measures the arterial pulsatile volume and reflects sympathetic nervous system augmentation due to desaturation during sleep. It has been validated against polysomnography [27]. Using specific signal patterns, a clinically validated algorithm provides indices used for determining the degree of sleep apnea. OSA was diagnosed if the apnea-hypopnea index (AHI) was ≥5. Moderate-to-severe OSA was defined as AHI ≥ 15. Oxygen desaturation index (ODI) is the number of times per hour of sleep that the oxygen level decreases by at least 3% from baseline.

### 2.3. Statistical Analysis

Data are presented as the mean ± standard deviation (SD), median with interquartile range (IQR), or frequency with percent. Mann–Whitney *U* test and independent *t*-test were used to compare continuous variables between those with and without any diabetes-related complications. The chi-square test was used to compare categorical variables.

To investigate whether OSA had direct effects on diabetic complications or whether this was indirectly mediated by the presence of hypertension, mediation analysis was performed. A mediation analysis is a standard statistical method which attempts to identify whether the relationship between an independent variable and an outcome variable is mediated by a third, or “mediator,” variable [28]. In this study, the presence of moderate-to-severe OSA (AHI ≥ 15) was an independent variable, hypertension was a mediator, and any diabetic complications were an outcome variable. First, a mediation model was constructed by fitting moderate-to-severe OSA as an independent variable on hypertension, adjusting for covariables including age, sex, BMI, diabetes duration, HbA1c, and statin use. Second, an outcome model was constructed by fitting any diabetes-related complications on hypertension and moderate-to-severe OSA, adjusting for covariables as previously described. Only significant covariables were kept in each mediation and outcome model. An indirect effect of moderate-to-severe OSA on any diabetes-related complications through hypertension was then estimated by multiplying coefficients of OSA in the mediation model with the coefficient of hypertension in the outcome model. A bootstrap of 1000 replications was then applied to the estimated indirect effect and its 95% confidence interval (CI). These analyses were performed using a generalized structural equation model, with a family of Bernoulli distributions and logit link function for both mediation and outcome models. All analyses were done using Stata statistical software version 14.0.

To determine if OSA severity was associated with eGFR, the following analyses were performed. Univariate linear regression analysis was used to determine the association between baseline characteristics, HbA1c, hypertension, ACEI/ARB use, sleep parameters (AHI and ODI), and eGFR. Stepwise backward regression analysis, adjusting for age, BMI, HbA1c, diabetes duration, hypertension, ACEI/ARB use, and statin use, was utilized to determine if AHI or ODI was an independent predictor of eGFR. For these analyses, natural logarithm-transformed values (Ln) of HbA1c, diabetes duration, eGFR, AHI, and ODI were used, since they were not normally distributed; *p* values < 0.05 were considered statistically significant.

## 3. Results

A total of 131 patients participated in the study. Their demographic and glycemic characteristics, diabetes-related complications, and sleep parameters are shown in Table 1. The mean age was 52.6 years, 55% were female, and median HbA1c was 7.4%. Forty-eight (37.2%) participants had DKD, 25 (19.5%) had retinopathy, 30 (23.3%) reported neuropathy symptoms, and overall 71 (55.5%) participants had at least one diabetes-related complication. Median (IQR) eGFR was 94.5 mL/min/1.73 m^2^ (79.6, 104.6). Over three-quarters of the participants had OSA, and 40.5% had moderate-to-severe OSA, with an overall median AHI (IQR) of 10.9 (5.2, 21.3).

### 3.1. OSA, Diabetes-Related Complications, and Hypertension

When categorizing the participants into those with or without any diabetes-related complications, it was found that those with any complications had poorer glycemic control and were more likely to have hypertension and be receiving treatment with ACEI/ARB (Table 2). In addition, they had more severe OSA, as indicated by higher median AHI and ODI, and were more likely to be diagnosed with OSA than those without complications. More of those with any complications had moderate-to-severe OSA than those without complications (45.1% versus 35.1%), although this did not reach statistically significant levels.

Because those with hypertension were more likely to have diabetes-related complications and it is known that OSA can lead to hypertension, we explored further by categorizing participants according to their diagnosis of hypertension (Table 2). This revealed that those with hypertension were older, had higher BMI, were more likely to be using ACEI/ARB, and had more severe OSA than those without hypertension. More hypertensive patients also had moderate-to-severe OSA than those without hypertension (*p* = 0.002).

### 3.2. Hypertension as a Mediator between OSA and Diabetes-Related Complications

Since those with any diabetes-related complications had more severe OSA and were more likely to have hypertension and hypertension was strongly associated with OSA, we further explored if having hypertension could be a potential mediator between OSA and diabetes-related complications.

A mediation model was constructed by fitting OSA on hypertension, adjusting for covariables including age, BMI, and diabetes duration. This model indicated that T2DM patients with concurrent moderate-to-severe OSA were about 2.9 times more likely to have hypertension than those with mild or no OSA (odds ratio calculated as *e^B^* = *e*^1.074^; Table 3). A diabetes-related complication outcome model was then constructed. None of the covariables were significantly associated with diabetes-related complications; thus, only OSA and hypertension were included in this model. This indicated that patients with hypertension were 2.8 times more likely to have any diabetes-related complications than those without hypertension.

**Figure 1:**
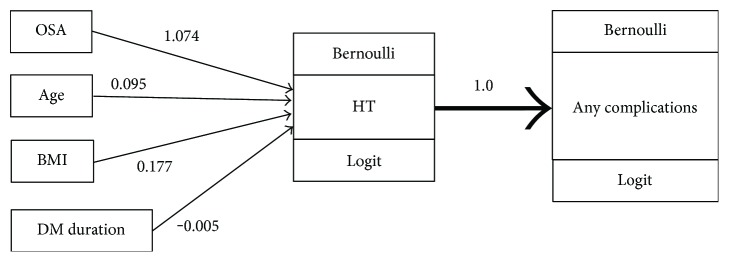
A mediation diagram of moderate-to-severe OSA➔hypertension (HT)➔any diabetes-related complications. Bernoulli distribution was regressed on OSA, adjusting for age, BMI, and DM duration, with logit link. An “any diabetes-related complications” model was constructed with similar distribution and link function.

A bootstrap with 1000 replications was applied and revealed that T2DM patients with concurrent moderate-to-severe OSA would have an odds ratio of having any diabetes-related complications, mediated through having hypertension, of 3.05 (95% CI: 1.03, 25.78) (Table 4 and Figure 1), while OSA had no direct effect on complications. This suggested that OSA was associated with hypertension, which in turn increased the risk of having diabetes-related complications.

### 3.3. OSA Severity and eGFR

The association between OSA severity and eGFR was further explored.  Table 5 demonstrates the results of univariate linear regression analysis between demographic, glycemic, and sleep parameters and eGFR. Older age, longer diabetes duration, having hypertension, and statin or ACEI/ARB use were associated with lower eGFR. In addition, more severe OSA, as indicated by higher AHI and ODI, was significantly associated with lower eGFR.

To determine if OSA severity was an independent predictor of eGFR, a stepwise backward regression analysis, adjusting for age, BMI, diabetes duration, HbA1c, hypertension, ACEI/ARB use, and statin use, was performed (Table 6). This demonstrated that higher AHI was borderline associated with lower eGFR (*B* = −0.037, *p* = 0.063), while higher ODI was significantly associated with lower eGFR (*B* = −0.036, *p* = 0.041).

## 4. Discussion

In this study of T2DM patients, the results revealed that OSA was associated with diabetes-related complications, and this relationship was mediated by the presence of hypertension. The mediation analysis suggested that T2DM patients with moderate-to-severe OSA and hypertension were three times more likely to have diabetes-related complications compared to those with no or mild OSA. In addition, the degree of intermittent hypoxia (ODI) was found to be independently associated with lower eGFR in this group of patients without ESRD, after adjusting for multiple confounders including hypertensive status. Collectively, these data support the role of OSA in diabetes-related complications and that hypertension plays at least a partial role in these relationships.

The data from this current study are in agreement with several previous studies demonstrating the effects of OSA on diabetic micro- and macrovascular complications. For example, a longitudinal study in 132 T2DM patients, followed for 4.9 years, revealed that sleep-disordered breathing was a predictor of incident coronary artery disease with a hazard ratio of 1.9 (95% CI: 1.1, 3.3), as well as an increased risk for heart failure [29]. In the Sleep AHEAD study which included 305 T2DM patients, AHI was associated with a 2.57-fold increase in risk of having a history of stroke [30]. Similar associations were found for microvascular complications. While a systematic review of eight studies found no convincing evidence that OSA was associated with diabetic retinopathy [17], a more recent longitudinal study revealed that OSA was a risk factor for progression to advanced diabetic retinopathy (odds ratio [OR], 5.2; 95% CI: 1.2, 23.0) [18]. A meta-analysis of five studies (880 patients) demonstrated an association between OSA and diabetic neuropathy (a pooled OR of 1.90), with a higher risk seen in those younger than 60 [31]. A more detailed assessment by skin biopsies from 52 T2DM patients revealed that higher AHI was associated with lower intraepidermal nerve fiber density, suggesting small fiber neuropathy [19]. The data from this current study suggested that hypertension plays an important role in the association between OSA and diabetes-related complications seen in this cohort. This is not surprising, as OSA is associated with hypertension, and CPAP treatment was shown to be beneficial in those with resistant hypertension [32]. Tighter blood pressure control in T2DM (144/82 versus 154/87 mmHg) was shown to reduce the risk of retinopathy and albuminuria [33], although lowering blood pressure to a target systolic of <120 mmHg did not further reduce the progression of retinopathy [34].

Only a few studies have explored the relationship between eGFR and OSA severity exclusively in T2DM. A cross-sectional study in 90 participants by Leong et al. found that AHI and time spent under O_2_ saturation of 90% were negatively correlated with eGFR, suggesting the adverse effects of hypoxemia [35]. This was confirmed in a larger study of 880 hospitalized patients in China, which demonstrated a negative association between ODI and eGFR [36]. In addition, a cohort study with a follow-up of 2.5 years revealed that patients with T2DM and OSA had a faster decline in their eGFR than those without OSA, with baseline nitrosative stress being an independent predictor of eGFR decline [37]. The current results are in agreement with these previous findings and underscore the importance of intermittent hypoxia as ODI, but not AHI, related to lower eGFR. In addition, patients with ESRD were excluded from the current study, and a majority of the patients (89.3%) had eGFR ≥ 60 mL/min/1.73 m^2^; thus, the relationship between OSA and renal function exists even in the early stages of DKD.

Several potential mechanistic pathways could explain the link between OSA and diabetes-related complications. Intermittent hypoxemia leads to vasoconstriction and increased oxidative stress, such as alterations in nitric oxide synthase activity, resulting in endothelial dysfunction and microvascular impairment [20]. Increased sympathetic nervous system activity, increased endothelin-1 levels, and alterations in renin-angiotensin-aldosterone regulation could also contribute to elevated blood pressure [38], and the latter could mediate the relationship between OSA and DKD. Nighttime hypoxemia was found to lead to an increase in vascular endothelial growth factor (VEGF) [39], a hypoxia-sensitive glycoprotein stimulating neoangiogenesis, which has been shown to play a role in diabetic retinopathy [40]. The OSA-related increase of inflammatory markers and hypercoagulability are also likely factors [20, 41]. In addition, OSA can impact glycemic control in T2DM, although this relationship was not found in the current cohort (data not shown). Increased advanced glycation end products and alterations in protein kinase C signaling are common pathways by which OSA and hyperglycemia contribute to microvascular complications [42, 43]. Through these multiple pathways, OSA could exert adverse effects on diabetes-related complications.

These data suggested that CPAP could be beneficial in reducing diabetes-related complications. While there is evidence supporting the favorable effects on blood pressure in T2DM [44, 45], no randomized trials exploring the effects of CPAP on diabetes-related complications have been conducted. Thus, current data were mostly observational. In a small cohort of 47 patients, those who were compliant with CPAP therapy had a slower decline of their eGFR after 2.5 years [37]. In another study in 38 T2DM patients, those who were compliant with CPAP were less likely to progress to advanced retinopathy [18]. As CPAP treatment was shown to downregulate renin-angiotensin-aldosterone activity [46] and reduce VEGF levels [47] in those without diabetes, these could possibly be mechanistic pathways by which CPAP use is associated with reduced risk of DKD and retinopathy. No data are available with regard to neuropathy. Thus, further research in this area is warranted, as OSA is very prevalent in the T2DM population and measures are needed to reduce morbidity and mortality related to diabetic complications.

The strength of this study was a comprehensive assessment of diabetes-related complications by applying mediation analysis. The exclusion of patients with ESRD revealed the relationship between OSA and eGFR even in patients with relatively normal renal function. However, there are limitations. The cross-sectional design precludes an assumption of a causal relationship. Importantly, there could be differing relationships between OSA and diabetes-related complications at different blood pressure levels or circadian variations. There is evidence that OSA is associated with a nondipping pattern of nocturnal blood pressure [48, 49], which has been shown to be associated with albuminuria and reduced eGFR in T2DM [50], as well as increased all-cause mortality [51]. Lack of comprehensive blood pressure measurements, especially in a longitudinal manner or with 24-hour monitoring, did not allow us to evaluate this possible relationship. Moreover, the compliance with antihypertensive therapies was not available. In this study, only moderate-to-severe OSA was used in the mediation analysis, as AHI or AHI ≥ 5 was not found to be associated with diabetes-related complications after adjusting for hypertension (data not shown). This, however, was consistent with evidence of a dose-response relationship between OSA severity and adverse health outcomes [23]. In addition, standard polysomnography was not used in this study, and WatchPAT could not differentiate central from obstructive events. However, we excluded those with risk factors for central apnea, as described above. Because of this, more severe diabetes-related complications such as stroke and heart failure were not evaluated in this study, even though these were reported to be associated with OSA in the general population [52, 53]. Lastly, the overall sample size of 131 may be relatively small to detect associations in few causal pathways of mediation analysis. As for estimations of post hoc power of the test, the powers were 73%, 93.3%, and 52.1% for OSA➔hypertension, hypertension➔diabetes-related complications, and OSA➔diabetes-related complications, respectively.

In conclusion, OSA, especially moderate-to-severe degree, was associated with diabetes-related complications in T2DM. This relationship was mediated by hypertensive state. In addition, more severe intermittent hypoxemia was related to lower eGFR, independently of hypertension, in this patient group with relatively normal renal function. Further studies exploring the effects of CPAP on diabetes-related complications are warranted.

## Figures and Tables

**Table 1:** Baseline characteristics of all participants.

	*n* = 131
*Demographic and glycemic characteristics*	
Age (years)	52.6 (11.5)
Sex (female)	72 (55.0%)
BMI (kg/m^2^)	28.8 (5.1)
Neck circumference (cm)	37.7 (3.9)
Diabetes duration (years)	10.3 (8.7)
HbA1c (%)	7.43 (6.68, 8.74)
Hypertension	92 (70.2%)
ACEI or ARB use	72 (55.0%)
Statin use	104 (79.4%)
eGFR (mL/min/1.73 m^2^)	94.5 (79.6, 104.6)
Serum creatinine (mg/dL)	0.87 (0.37)
*Diabetic complications*	
Diabetic nephropathy	48 (37.2%)
Diabetic retinopathy	25 (19.5%)
Neuropathy	30 (23.3%)
Coronary artery disease	7 (5.3%)
Any complications	71 (55.5%)
*Sleep parameters*	
AHI	10.9 (5.2, 21.3)
ODI	6.6 (2.0, 14.0)
OSA diagnosis (AHI ≥ 5)	99 (75.6%)
AHI ≥ 15	53 (40.5%)

Data are presented as mean (SD), median (IQR), or number (%). ACEI: angiotensin-converting enzyme inhibitor; ARB: angiotensin receptor blocker; AHI: apnea hypopnea index; eGFR: estimated glomerular filtration rate; HbA1c: hemoglobin A1c; ODI: oxygen desaturation index.

**Table 2:** Comparisons between participants without and with any diabetes-related complications and those with and without hypertension.

	No complications (*n* = 57)	Presence of any diabetes complications (*n* = 71)	*p* value	Normotensive (*n* = 39)	Hypertensive (*n* = 92)	*p* value
*Demographic and glycemic characteristics*						
Age (years)	51.2 (10.9)	53.8 (11.5)	0.189	47.3 (13.0)	54.8 (10.0)	0.001
Sex (female)	32 (56.1%)	39 (54.9%)	0.891	25 (64.1%)	47 (51.1%)	0.171
BMI (kg/m^2^)	27.8 (5.1)	29.3 (4.9)	0.100	27.2 (4.7)	29.4 (5.1)	0.023
Diabetes duration (years)	9.4 (9.3)	11.2 (8.5)	0.250	8.5 (8.4)	11.0 (8.9)	0.141
HbA1c (%)	7.2 (6.6, 7.8)	7.9 (6.7, 9.5)	0.023	7.8 (6.6, 9.8)	7.3 (6.7, 8.6)	0.350
Hypertension	33 (57.9%)	57 (80.3%)	0.006	—	—	—
ACEI/ARB use	26 (45.6%)	45 (63.4%)	0.044	1 (2.5%)	71 (77.2%)	<0.001
Statin use	43 (75.4%)	59 (83.1%)	0.284	27 (69.2%)	77 (83.7%)	0.061
*Sleep parameters*						
AHI	8.8 (3.2, 17.3)	12.9 (7.3, 26.7)	0.013	6.1 (1.7, 14.5)	14.6 (8.0, 28.4)	<0.001
ODI	4.8 (1.2, 10.6)	8.3 (3.6, 18.2)	0.018	2.6 (0.6, 8.0)	8.4 (3.8,18.9)	<0.001
AHI ≥ 5	38 (66.6%)	58 (81.7%)	0.051	23 (58.9%)	76 (82.6%)	0.004
AHI ≥ 15	20 (35.1%)	32 (45.1%)	0.253	8 (20.5%)	45 (48.9%)	0.002

Data are presented as mean (SD), median (IQR), or number (%). ACEI: angiotensin-converting enzyme inhibitor; ARB: angiotensin receptor blocker; AHI: apnea hypopnea index; eGFR: estimated glomerular filtration rate; HbA1c: hemoglobin A1c; ODI: oxygen desaturation index.

**Table 3:** Mediation models of moderate-to-severe OSA➔hypertension (HT)➔any diabetes-related complications.

Model	Factors	*B*	SE	*t*	*p*	95% CI
Moderate-to-severe OSA➔HT	OSA	1.074	0.488	2.201	0.028	0.118, 2.031
	Age	0.095	0.025	3.806	0.000	0.046, 0.144
	BMI	0.177	0.055	3.229	0.001	0.069, 0.284
	Diabetes duration	−0.005	0.031	−0.158	0.874	−0.065, 0.055
HT➔any complications	HT	1.038	0.414	2.509	0.012	0.227, 1.849
	OSA	0.177	0.386	0.458	0.647	−0.580, 0.934

*B*: unstandardized coefficient; SE: standard error; *t* = *t*-test statistic.

**Table 4:** Estimation of mediation effects in mediation pathway of moderate-to-severe OSA➔hypertension (HT)➔any diabetes-related complications.

	Pathway	Odds ratio	95% CI
Indirect	OSA➔HT➔any complications	3.049	1.026, 25.775
Direct	OSA➔any complications	1.194	0.546, 2.654

**Table 5:** Univariate linear regression analysis to evaluate associations between baseline characteristics, sleep parameters, and Ln eGFR.

Variables	*B*	*p* value
Age	−0.016	<0.001
Sex	0.093	0.114
BMI	0.011	0.064
Ln diabetes duration	−0.087	<0.001
Ln HbA1c	0.313	0.038
Hypertension	−0.176	0.006
ACEI/ARB use	−0.115	0.049
Statin use	−0.133	0.066
Ln AHI	−0.057	0.016
Ln ODI	−0.050	0.014

*B*: unstandardized coefficient; ACEI: angiotensin-converting enzyme inhibitor; ARB: angiotensin receptor blocker; AHI: apnea hypopnea index; eGFR: estimated glomerular filtration rate; HbA1c: hemoglobin A1c; Ln: natural logarithm; ODI: oxygen desaturation index.

**Table 6:** Stepwise backward regression analysis to determine the independent predictors of Ln eGFR.

Variables	*B*	*p* value	*B*	*p* value
Age	−0.015	<0.001	−0.015	<0.001
Ln AHI	−0.037	0.063	—	—
Ln ODI			−0.036	0.041

AHI: apnea hypopnea index; eGFR: estimated glomerular filtration rate; Ln: natural logarithm; ODI: oxygen desaturation index.
